# Effect of closed-chain isometric plyometric training combined with palonosetron on postoperative knee function in patients after total knee arthroplasty

**DOI:** 10.3389/fsurg.2025.1512717

**Published:** 2025-03-21

**Authors:** Jingwen Shao, Shaoyi Qu, Jing Wang, Dan Sun, Qing Hu, Zhongxiang Luo

**Affiliations:** Department of Rehabilitation Medicine, Gongli Hospital of Shanghai Pudong New Area, Shanghai, China

**Keywords:** arthroplasty, replacement, knee, closed-chain isometric plyometrics, knee joint, muscle strength, walking

## Abstract

**Background:**

Functional training after total knee arthroplasty (TKA) is of great significance for the recovery of knee function. However, the utility of applying an effective training modality, closed-chain isokinetic plyometric training, on top of the drug palonosetron, which is used to prevent nausea and vomiting, is unclear.

**Objective:**

To investigate the effect of closed-chain isokinetic plyometric training combined with palonosetron-on-postoperative-knee-function in patients with TKA, aiming to provide an effective rehabilitation program for patients with TKA.

**Methods:**

The results of the intervention were compared in 47 TKA patients who received closed-chain isokinetic plyometric training combined with palonosetron with 47 TKA patients who received conventional plyometric training combined with palonosetron. Knee function was evaluated using the Hospital for Specialty Surgery Knee (HSS), quadriceps muscle strength was assessed using real-time isometric plyometric testing with the BIODEX-sYs-tem 4-type system, walking ability was evaluated using the Timed Up-and-Go Test (TUGT), and proprioception was determined using the Biodex System 4-type multi-joint isometric system.

**Results:**

After-the intervention,-the-HSS scores-of-the-experimental-group-were-higher-than-those-of-the-control-group-(*P* < 0.05); the peak moment values of the quadriceps (PT), the total quadriceps work values (STW), and the average power of the quadriceps (AP) were higher than those of the control group (*P* < 0.05); and the values of the reproduced deviation of the knee joint active joint angle and the TUGT time were lower than those of the control group (*P* < 0.05).

**Conclusion:**

Closed-chain isokinetic plyometric training combined with palonosetron can strengthen their muscle strength and improve proprioception, which effectively promotes the recovery of postoperative knee function and walking ability in TKA patients.

## 1 Introduction

Knee osteoarthritis (KOA) is a chronic degenerative osteoarthritic disease, which is prevalent in middle-aged and elderly people, and clinically manifested by deformation of articular cartilage, osteophytes, etc., which cause symptoms such as joint pain and limitation of movement (1). Total knee arthroplasty (TKA) is the main treatment for osteoarthritis of the knee, which can effectively improve dysfunction and relieve pain symptoms. Against the backdrop of rising global obesity rates and an accelerating aging population, KOA is on the rise year after year, and the number of TKA clinics will continue to increase (2). Although TKA has shown positive therapeutic outcomes, patients suffer from loss of knee muscle strength and function for months after surgery, which increases the risk of falls and leads to a loss of independence (3, 4). Therefore, effective plyometric exercises are needed to promote the recovery of muscle strength and function after TKA.

Isokinetic technique is a safe and effective method of muscle training. Isokinetic plyometrics allows the limb to move at a predetermined angular velocity, which does not change with muscle tension, promotes stretching or shortening of muscle fibers, and improves the performance of the muscle cells to enhance muscle strength (5). Isokinetic exercise has been shown to maximize muscle strength and enhance muscle performance (6). In isokinetic exercise, since individual joint training may cause joint involvement, compound training that mobilizes multiple joints, known as chaining, is often used. Among them, the open chain mode refers to the proximal limb fixation while moving the distal joint, and the closed chain mode refers to the distal limb fixation while moving the proximal joint. It has been shown that closed chain exercise can effectively improve the stability of the joint, can promote the recovery of the knee joint function, and at the same time can effectively prevent the decline of other functions of the affected limb (7–9). However, previous studies have applied closed-chain isokinetic plyometrics to postoperative anterior cruciate ligament injuries of the knee (10) and outpatient treatment of osteoarthritis of the knee (11), and the effect of postoperative closed-chain isokinetic plyometrics after TKA has not yet been reported.

The use of anesthetic drugs during TKA can cause severe postoperative nausea and vomiting (PONV) (12). The occurrence of nausea and vomiting will lead to the inability of KOA patients to carry out normal muscle training after surgery, hindering the recovery of muscle strength and joint function. Therefore, prevention and treatment of PONV are needed to ensure normal postoperative muscle training. The incidence of PONV after lower extremity total joint arthroplasty without prophylactic antiemetic drugs is reported to be 68%–83% (13). PONV may lead to the inability of patients to carry out normal functional exercises after surgery, increase the incidence of thrombosis, and prolong the length of hospitalization (14). Palonosetron is the newest 5-HT3 receptor antagonist and has the advantage of a longer half-life and greater receptor affinity than other antagonists (15). Palonosetron has been shown to be effective in reducing the incidence of nausea and vomiting after TKA (16, 17).

At present, no significant results have been achieved in the clinic for rehabilitation training after TKA surgery, and traditional rehabilitation training is difficult to restore the patient's knee function to a normal state in a short period of time. Therefore, we conducted a randomized controlled trial aiming to investigate the effect of closed-chain isokinetic muscle training combined with palonosetron in patients undergoing total knee arthroplasty and to provide a reference for postoperative rehabilitation of TKA patients.

## 2 Methods

### 2.1 Experimental preparation

Ninety-four patients were selected to undergo TKA in our hospital from August 2021 to January 2023, and were divided into 47 patients each in the control group (traditional plyometrics combined with palonosetron) and the experimental group (closed-chain isometrics combined with palonosetron) by applying the random number table method. All patients signed an informed consent form, and the study passed the ethical review of our hospital.

### 2.2 Inclusion and exclusion criteria

Inclusion criteria: (1) those who intend to perform TKA with surgical indications; (2) ASA classification I-III; (3) high compliance; (4) The same prosthesis was used in both groups and the posterior cruciate ligament was preserved. Exclusion criteria: (1) those with osteoporosis and bone tumors; (2) those with organ dysfunction; (3) those with previous history of knee surgery; (4) those with cognitive disorders; (5) those with coagulation disorders.

### 2.3 Treatment

In both groups, palonosetron [Shanghai Huayuan Pharmaceutical (Ningxia) Shasai Pharmaceutical Co., Ltd; China National Drug Code H20080748; 5 ml: 0.25 mg] was intravenously injected 1.5 ml before anesthesia induction, and the injection time was >10 s.

The control group received traditional muscle strength training, and the affected limbs began training after surgery when they could be tested for quadriceps muscle strength (they could move between the bed and chair and get up on their own). The muscle strength test method: the patient was lying down, referring to the lower limb muscle resistance training, to determine the maximum muscle strength of the knee joint at 45° long knee extension and knee flexion, and the result was the quadriceps and hamstring muscle strength, the relevant movements should be stabilized for 10 s during the test period, 10 consecutive tests, and the average value was calculated. A training program was developed by combining the results of the muscle strength test. In the early postoperative period, the training program was mainly based on slow resistance, and the difficulty of resistance training and the difficulty of standing and walking were used to develop the difficulty and intensity of the next training program, which lasted for 8 weeks. (1) Within 3 days after surgery, the patient takes the supine position, the foot is kept at 90° to the bed surface, the foot is hooked up with force, and then stepped down with force, and each movement is insisted for 3–5 s. The healthy leg is bent, the affected leg is straight, the foot of the affected side is at 90° to the bed surface, the affected side tenses the thigh muscles and insists for 3–5 s, then relaxes. (2) 4–7 days after surgery, family members hold the patient's knee with one hand, hold the ankle with the other hand, the patient's whole body relaxes, and do flexion and extension exercises with the assistance of family members. When the range of knee flexion and extension reaches a level that allows the patient to hold his/her hands below the knee, then the patient will perform knee flexion and hip flexion exercises by himself/herself. Hold each movement for 3 s and do 10 strokes every 1 h. (3) 1–2 weeks after the operation, when the patient can move freely in bed with the knee flexed, and when there is no pain in the injured leg with weight bearing, the patient can get out of bed and walk around on his/her own (with the help of crutches).

The experimental group received closed-chain isokinetic muscle training in the following ways: (1) 2–3 weeks after surgery, start quadriceps contraction, ankle pump training, every 5 s, bedside passive flexion and extension training, 3 times a day. (2) 3–5 weeks postoperatively, start supine knee flexion activity, the heel needs to be kept on the bed during the training period, the angle of knee flexion <30°, the training time is half an hour, the frequency is 3°/s, twice a day, 6 days a week, and the cold compress is applied for half an hour after the training. (3) 6–8 weeks after the operation, instruct the patients to use single crutches for weight-bearing training, and exercise semi-squatting movement, with the angle of knee flexion <45°. (4) After 8 weeks postoperatively, full weight-bearing training was carried out, including walking, half-squatting, balance board, and going up and down stairs. (5) Isokinetic closed chain training, using isokinetic muscle training system System4 to start training, the patient sitting position, to ensure stability, the affected limb is placed on the foot pedal to start isokinetic knee flexion and extension, the training rate of 60 s/10 times, 120 s/15 times, 180 s/20 times, each time 2 sets, 3∼4 times a week, training for 8 weeks.

### 2.4 Primary outcome

Knee function score: the knee function was evaluated before and after intervention using the Hospital for Specialty Surgery Knee (HSS), including six dimensions of pain, function, mobility, muscle strength, flexion deformity, and stability, with a score range of 0 to 100, and the higher the score, the better the function.

### 2.5 Secondary outcomes

Biological indicators of quadriceps muscle strength: real-time isometric muscle strength testing was performed before and after the intervention using a BIODEX-sYs-tem4 type system at an angle of 60°/s. Peak torque values of quadriceps muscle (PT), total quadriceps muscle work (STW), and quadriceps muscle average power (AP) were recorded, and the higher the value, the better the muscle strength.

Functional Walking Ability Score: Walking ability was evaluated using the Timed Up-and-Go Test (TUGT) before and after the intervention, recording the time of getting up, walking 3 meters, turning around, and walking back-to-sitting down for three repetitions in both groups, and calculating the mean, with the shorter time being the more functional.

Proprioception: The Biodex System 4 multi-joint isometric system was used to determine the active joint angles before and 8 weeks after the intervention. The patients were instructed to flex their knees at 30° and 60° from 90° of flexion for 10 s to familiarize themselves with the 2 positions, and then the patients were instructed to flex their knees at 30° and 60° from 90° of flexion and the angular positional deviation values were recorded, and the measurements were repeated for 3 times. The smaller the absolute deviation value, the better the proprioception.

### 2.6 Statistical processing

Use SPSS 26.0 software (IBM, USA) to process the data, the normal disreputability of the data was analyzed using the Shapiro-wilk test, conforming to the normal distribution of measurement data expressed by ($\bar{\chi }\pm s$), independent samples *t*-test differences between groups, paired *t*-test differences between before and after the same group, count data expressed by (%), x^2^ test differences; such as *P* < 0.05 suggests statistical significance.

## 3 Results

### 3.1 Demographics and baseline information

**Figure 1:**
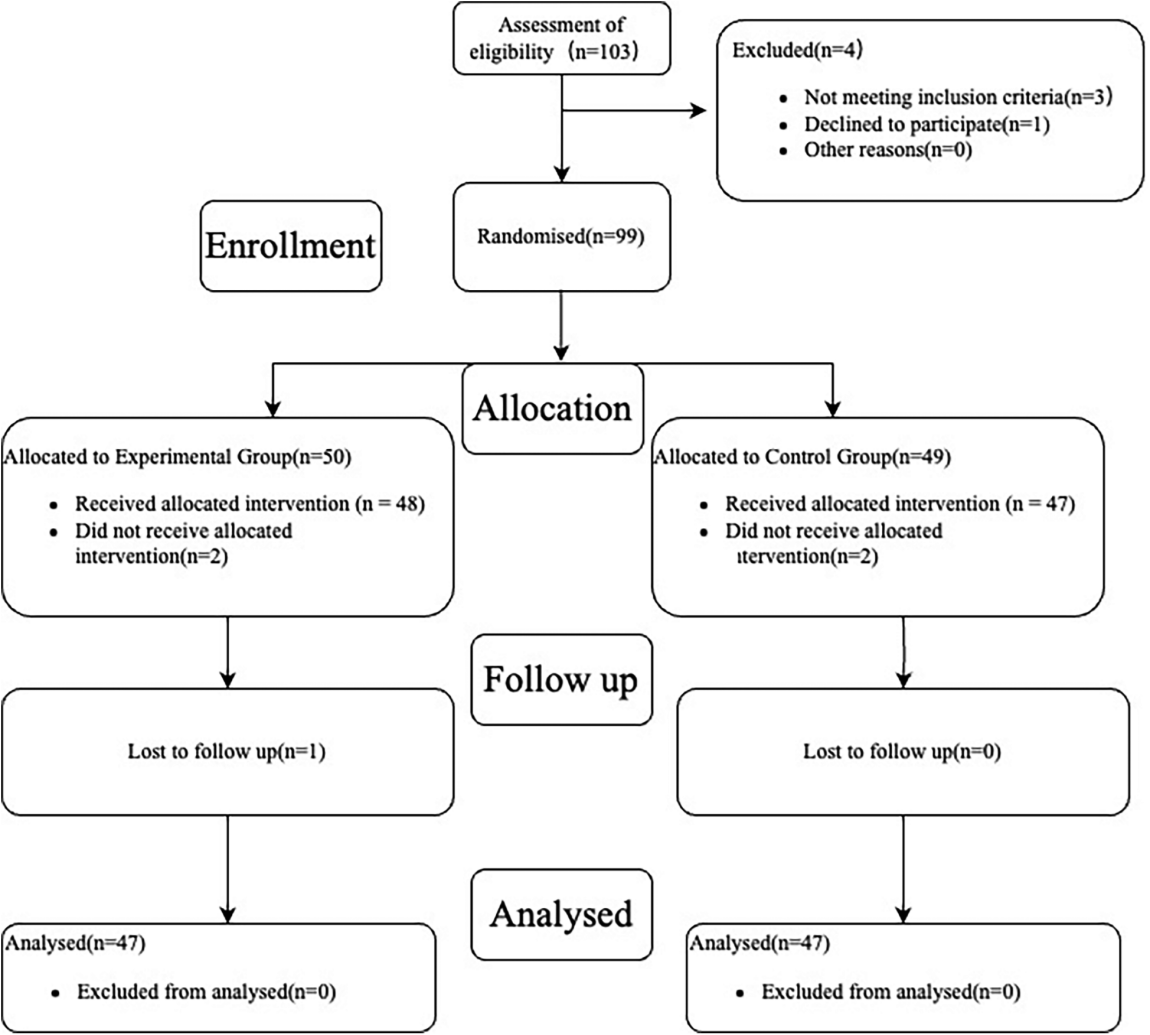
Process of inclusion of research subjects.

A total of 103 patients with KOA were enrolled in this study, 3 patients were excluded for not meeting the inclusion exclusion criteria and 1 patient refused to participate in the study. The remaining 99 patients were randomized into a trial group (*n* = 50) and a control group (*n* = 49). In the test group, 2 patients refused to accept the treatment modality to which they were assigned, and 1 patient was lost during the follow-up phase. In the control group, 2 patients refused to accept the assigned treatment modality and no patient was lost at the follow-up stage. No patients were excluded from the data analysis stage in either group. As shown in Figure 1.

As shown in Table 1, there was no statistically significant difference in demographic and baseline information between the two groups (*P* > 0.05).

**Table 1:** Demographics and baseline information of the two groups.

Variable		Experimental group (*n* = 43)	Control group (*n* = 43)	*X*^2^ ot *t*	*P* Value
Age, years, mean ± SD		63.15 ± 2.87	63.44 ± 2.91	0.486	0.628
Male/Female, *n* (%)		25 (53.19)/22 (46.81)	24 (51.06)/23 (48.94)	0.043	0,836
Affected limb, *n* (%)	Left	24 (51.06)	22 (46.81)	0.170	0.680
	Right	23 (48.94)	25 (53.19)		
BMI, kg/m^2^, mean ± SD		24.60 ± 1.19	24.81 ± 1.25	0.834	0.406
ASA	Ⅰ	15 (31.91)	14 (29.79)	0.305	0.859
	II	25 (53.19)	24 (51.06)		
	III	7 (14.89)	9 (19.15)		

### 3.2 Knee function score

Before the intervention, the difference between the HSS scores of the two groups was not statistically significant (*P* > 0.05); after the intervention, the HSS of both groups increased, and the experimental group was higher than the control group, and the difference was statistically significant (*P* < 0.05). See Table 2.

**Table 2:** Comparison of knee function scores between the two groups ($\bar{\chi }\pm s$).

Projects		Experimental group (*n* = 43)	Control group (*n* = 43)	*t*	*P*
HSS, scores	Before intervention	40.90 ± 2.19	40.56 ± 2.14	0.761	0.448
	After intervention	90.42 ± 4.90*	86.01 ± 4.69*	4.457	<0.001

* *P* < 0.05 compared to pre-intervention.

### 3.3 Biomechanical indices of the quadriceps muscle

Before intervention, the difference in quadriceps biomechanical indexes between the two groups was not statistically significant (*P* > 0.05); after intervention, quadriceps biomechanical indexes improved in both groups, and PT, STW, and AP of the experimental group were higher than those of the control group, and the difference was statistically significant (*P* < 0.001). See Table 3.

**Table 3:** Comparison of quadriceps muscle strength between the two groups (*n*, %).

Projects		Experimental group (*n* = 43)	Control group (*n* = 43)	*t*	*P*
PT (N/m)	Before intervention	50.44 ± 6.19	50.16 ± 6.12	0.220	0.826
	After intervention	82.81 ± 8.14*	62.03 ± 7.58*	12.808	<0.001
STW (J)	Before intervention	253.11 ± 21.30	252.38 ± 21.29	0.166	0.868
	After intervention	407.25 ± 29.61*	346.05 ± 25.85*	10.674	<0.001
AP (W)	Before intervention	31.58 ± 6.19	31.49 ± 6.14	0.071	0.944
	After intervention	45.33 ± 8.40*	39.12 ± 7.66*	3.745	<0.001

* *P* < 0.05 compared to before intervention.

### 3.4 Functional walking ability

Before the intervention, the difference in functional walking ability between the two groups was not statistically significant (*P* > 0.05); after the intervention, the TUGT time was shortened in both groups, and the experimental group was shorter than the control group, and the difference was statistically significant (*P* < 0.001). See Table 4.

**Table 4:** Comparison of functional walking ability between the two groups ($\bar{\chi }\pm s$).

Projects		Experimental group (*n* = 43)	Control group (*n* = 43)	*t*	*P*
TUGT (s)	Before intervention	50.12 ± 4.10	50.35 ± 4.01	0.275	0.784
	After intervention	15.64 ± 1.04*	19.51 ± 1.23*	16.471	<0.001

* *P* < 0.05 compared to before intervention.

### 3.5 Proprioception

Before the intervention, the difference between the reproduced deviation values of the active joint angles of the knee joints of the two groups was not statistically significant (*P* > 0.05); after the intervention, the reproduced deviation values of the active joint angles of the knee joints of the two groups were reduced, and the experimental group was lower than that of the control group, and the difference was statistically significant (*P* < 0.05). See Table 5.

**Table 5:** Comparison of proprioception between the two groups ($\bar{\chi }\pm s$, °).

Projects		Experimental group (*n* = 43)	Control group (*n* = 43)	*t*	*P*
30°	Before intervention	9.56 ± 2.64	9.46 ± 2.58	0.186	0.853
	After intervention	4.81 ± 1.10*	5.29 ± 1.23*	1.994	0.049
60°	Before intervention	9.01 ± 2.16	8.59 ± 2.12	0.951	0.344
	After intervention	3.19 ± 1.02*	3.65 ± 1.20*	2.002	0.048

* *P* < 0.05 compared to before intervention.

## 4 Discussion

The results of this study showed that closed-chain isokinetic plyometric training can effectively improve quadriceps muscle strength, improve proprioception, promote the recovery of knee function, and improve the walking ability of patients with KOA after KTA, and the overall effect is excellent.

TKA surgery is a very traumatic procedure for the patient, which can lead to inflammatory reaction in the body, induce severe disease and pain in the knee joint and its surrounding tissues, reduce the compliance of the soft tissues around the knee joint, increase the resistance to joint movement, and the lack of muscle strength is the main problem after TKA surgery (18). TKA surgery removes tissues that have proprioceptors such as articular bone, cruciate ligament, meniscus, which leads to a certain degree of reduction or complete loss of proprioception, and induces patients' balance ability, joint function, gait abnormality, etc. This leads to a certain degree of reduction or complete loss of proprioception, which induces balance, joint function reduction, gait abnormality, etc. (19). Postoperative balance disorder and walking abnormality in TKA patients can be caused by a variety of factors, including limb muscle weakness, psychological factors, especially abnormal input of sensory information (proprioception) (20). Therefore, rehabilitation training is needed after KTA to promote the recovery of knee function, but there is no standardized clinical training standard.

Since its introduction in the 1960s, isokinetic technology has been recognized as a revolution in plyometric testing and training (21). Isokinetic exercise provides a compliant resistance to the contraction of the patient's own muscles during the intervention, and isokinetic muscle contraction is characterized by isometric and isotonic contractions, while isokinetic training can lead to the quantification of the effects of the rehabilitation training (22). Isokinetic training is computerized, which can make up for the problem of insufficient or excessive training intensity of traditional training methods. Closed-chain isokinetic training changes the movement to linear movement without increasing the joint shear force, protects the knee joint, reduces the force load on the flexor and extensor muscles, effectively stimulates the proprioceptors, and restores the function of the proprioceptors (23).

The results of this paper show that the reproducible deviation value of the active joint angle of the proprioceptive knee joint of the experimental group is lower than that of the control group, and the biomechanical index of the quadriceps muscle is better than that of the control group; it suggests that closed-chain isokinetic plyometric training can improve the proprioceptive function and the quadriceps muscle strength. Proprioception refers to the body's active or passive motion sensation, mainly from the body joints, skin, muscles and other receptors are stimulated to obtain, can judge the body space, the size of the force (24). Proprioception includes motion sensation, position sensation, and vibration sensation, and the accuracy and completeness of proprioception depends on the proprioceptors in the muscle spindle and joints. During exercise, when the proprioceptors are stimulated, the center responds to the stimulus and activates the neural control function, prompting the motor units to participate in the acquisition. Traditional plyometric training focuses on muscle strength training around the knee joint, without training on proprioceptive infection (25). Relevant studies have confirmed that quadriceps muscle strength is closely related to proprioception, and muscle strength and proprioception have a mutually reinforcing effect (26). When patients perform closed-chain isokinetic muscle strength training, they can perform exercise training at a predetermined angle and speed, and do not change with muscle tension, which promotes effective stretching of muscle fibers, excites the nerves through neurological and biochemical regulatory pathways, activates LA-like afferent fibers in the Myo fasciculus, transmits motor information to the brain via the cortex, increases motor unit recruitment, enhances myosin mitochondrial enzyme activity, and then improves myocyte function. This structure can control the information output of the muscle shuttle, which significantly enhances the motor nerve activity of the muscle shuttle and strengthens the quadriceps muscle strength (8, 27, 28).

The results of this paper show that the HSS score of the experimental group is higher than that of the control group; it suggests that the closed-chain isokinetic plyometric training can improve the knee joint function after KTA. Compared with traditional muscle training, closed-chain isokinetic plyometric training provides patients with more stable angle and balanced speed during exercise, and during exercise training, the closed-chain isokinetic plyometric training system can provide patients with compliant resistance by combining with patients' muscle strength, muscle length, arm length, pain, etc. The amount of force required by the patients can be varied with the change of muscle tension, providing patients with continuous resistance training, and at the same time, the training has isotonic and isometric characteristics, prompting patients' muscles to reach the optimal training state, thus improving the function of KTA (29). With isotonic and isometric characteristics, it prompts the patient's muscles to reach the optimal training state, so it improves the peripheral muscle strength of the knee joint of the patients after KTA, and improves the joint stability, and promotes the recovery of the knee joint function.

The results of this paper showed that the TUGT time of the test group was shorter than that of the control group; suggesting that closed-chain isokinetic plyometric training can improve the walking ability of patients after KTA. Closed-chain isokinetic plyometric training is a linear movement of the three major joints combined, which can prompt the synergistic ability between the joints, and is in line with the principle of the organism's neurodevelopment from the hip to the foot (30). The repeated flexion and extension movements of the joints in the closed-chain mode need to recruit more neural unit signals to induce the contraction of the active and antagonist muscles, which is conducive to improving the stability of the affected limbs and improving the ability to walk. Limb dysfunction after KTA is associated with a decrease in the input of proprioceptive signals (31). Closed chain isometric training focuses on the training of the three major joints, strengthens the strength of the limb muscle groups, stimulates the joint and positional senses of the limbs, promotes the recovery of proprioception in the lower limbs, and enhances the neuromuscular control of the limbs.

The small number of cases included in this study may lead to some limitations in the findings. Meanwhile, the observation time of this study is relatively short, and it remains to be further observed whether closed-chain isokinetic muscle training combined with palonosetron has a favorable effect on long-term functional recovery in patients after total knee arthroplasty.

## 5 Conclusion

Closed-chain isokinetic muscle training can improve quadriceps muscle strength, proprioception, knee function, and walking ability of postoperative KTA patients, which has certain clinical value. However, more research is needed to confirm whether it can be widely promoted and applied in the clinic.

## Data Availability

The raw data supporting the conclusions of this article will be made available by the authors, without undue reservation.
